# Supplementary material for: Modeling glioblastoma heterogeneity as a dynamic network of cell states
Source: Mol Syst Biol. 2021 Sep 16;17(9):e10105. doi: 10.15252/msb.202010105 (PMC8444284; doi:10.15252/msb.202010105)
Supplement: Supplementary file 6 — Source Data for Figure 5 [file MSB-17-e10105-s004.zip › Figure5A_sourcedata/GSEA_3071/hallmarks_stateIV.GseaPreranked.1623742551702/HALLMARK_HYPOXIA.html]

Details for gene set HALLMARK\_HYPOXIA[GSEA]

|  || Dataset | state53071 |
| Phenotype | NoPhenotypeAvailable |
| Upregulated in class | na\_neg |
| GeneSet | HALLMARK\_HYPOXIA |
| Enrichment Score (ES) | -0.38462362 |
| Normalized Enrichment Score (NES) | -2.0528188 |
| Nominal p-value | 0.0 |
| FDR q-value | 9.972607E-4 |
| FWER p-Value | 0.008 |
Table: GSEA Results Summary

  

Fig 1: Enrichment plot: HALLMARK\_HYPOXIA      
 Profile of the Running ES Score & Positions of GeneSet Members on the Rank Ordered List

  

| PROBE | GENE SYMBOL | GENE\_TITLE | RANK IN GENE LIST | RANK METRIC SCORE | RUNNING ES | CORE ENRICHMENT || 1 | MAP3K1 |  |  | 42 | 1.903 | 0.0126 | No |
| 2 | IGFBP3 |  |  | 59 | 1.794 | 0.0290 | No |
| 3 | PDGFB |  |  | 86 | 1.615 | 0.0416 | No |
| 4 | STC1 |  |  | 97 | 1.547 | 0.0564 | No |
| 5 | NEDD4L |  |  | 100 | 1.535 | 0.0727 | No |
| 6 | CSRP2 |  |  | 175 | 1.305 | 0.0727 | No |
| 7 | NCAN |  |  | 181 | 1.298 | 0.0858 | No |
| 8 | CXCR4 |  |  | 197 | 1.269 | 0.0967 | No |
| 9 | FOXO3 |  |  | 291 | 1.054 | 0.0902 | No |
| 10 | HEXA |  |  | 315 | 1.018 | 0.0969 | No |
| 11 | KIF5A |  |  | 358 | 0.961 | 0.0992 | No |
| 12 | CDKN1B |  |  | 374 | 0.939 | 0.1065 | No |
| 13 | KLHL24 |  |  | 419 | 0.885 | 0.1077 | No |
| 14 | PAM |  |  | 431 | 0.871 | 0.1150 | No |
| 15 | PNRC1 |  |  | 481 | 0.830 | 0.1146 | No |
| 16 | FOS |  |  | 530 | 0.786 | 0.1139 | No |
| 17 | CDKN1C |  |  | 565 | 0.751 | 0.1155 | No |
| 18 | SDC2 |  |  | 571 | 0.747 | 0.1226 | No |
| 19 | WSB1 |  |  | 578 | 0.741 | 0.1295 | No |
| 20 | CCNG2 |  |  | 593 | 0.731 | 0.1348 | No |
| 21 | EXT1 |  |  | 744 | 0.636 | 0.1128 | No |
| 22 | VHL |  |  | 788 | 0.609 | 0.1111 | No |
| 23 | SCARB1 |  |  | 804 | 0.601 | 0.1147 | No |
| 24 | HK1 |  |  | 874 | 0.571 | 0.1076 | No |
| 25 | MXI1 |  |  | 944 | 0.546 | 0.1003 | No |
| 26 | STC2 |  |  | 972 | 0.535 | 0.1009 | No |
| 27 | VEGFA |  |  | 1010 | 0.518 | 0.0994 | No |
| 28 | RORA |  |  | 1060 | 0.503 | 0.0954 | No |
| 29 | CA12 |  |  | 1106 | 0.490 | 0.0920 | No |
| 30 | BTG1 |  |  | 1123 | 0.486 | 0.0942 | No |
| 31 | ZNF292 |  |  | 1153 | 0.479 | 0.0938 | No |
| 32 | CHST3 |  |  | 1185 | 0.470 | 0.0930 | No |
| 33 | PRKCA |  |  | 1214 | 0.461 | 0.0926 | No |
| 34 | IRS2 |  |  | 1218 | 0.458 | 0.0970 | No |
| 35 | CASP6 |  |  | 1265 | 0.450 | 0.0930 | No |
| 36 | GPC4 |  |  | 1269 | 0.448 | 0.0973 | No |
| 37 | ALDOC |  |  | 1284 | 0.443 | 0.0994 | No |
| 38 | SLC6A6 |  |  | 1684 | 0.353 | 0.0263 | No |
| 39 | VLDLR |  |  | 1866 | 0.319 | -0.0051 | No |
| 40 | SAP30 |  |  | 1914 | 0.311 | -0.0108 | No |
| 41 | KDM3A |  |  | 1981 | 0.301 | -0.0203 | No |
| 42 | DPYSL4 |  |  | 2060 | 0.288 | -0.0322 | No |
| 43 | ETS1 |  |  | 2305 | 0.254 | -0.0765 | No |
| 44 | GCNT2 |  |  | 2393 | -0.258 | -0.0904 | No |
| 45 | PGM2 |  |  | 2504 | -0.270 | -0.1087 | No |
| 46 | PDK1 |  |  | 2754 | -0.301 | -0.1535 | No |
| 47 | NAGK |  |  | 2759 | -0.301 | -0.1510 | No |
| 48 | JUN |  |  | 2785 | -0.304 | -0.1525 | No |
| 49 | JMJD6 |  |  | 2826 | -0.309 | -0.1568 | No |
| 50 | NOCT |  |  | 2835 | -0.310 | -0.1550 | No |
| 51 | GBE1 |  |  | 2881 | -0.317 | -0.1602 | No |
| 52 | BCL2 |  |  | 2901 | -0.319 | -0.1604 | No |
| 53 | ATF3 |  |  | 2977 | -0.330 | -0.1713 | No |
| 54 | TNFAIP3 |  |  | 3248 | -0.368 | -0.2194 | No |
| 55 | PGK1 |  |  | 3257 | -0.369 | -0.2169 | No |
| 56 | P4HA1 |  |  | 3355 | -0.382 | -0.2315 | No |
| 57 | HMOX1 |  |  | 3447 | -0.399 | -0.2447 | No |
| 58 | SDC3 |  |  | 3547 | -0.419 | -0.2592 | No |
| 59 | HSPA5 |  |  | 3555 | -0.419 | -0.2560 | No |
| 60 | MAFF |  |  | 3620 | -0.432 | -0.2636 | No |
| 61 | PPP1R15A |  |  | 3793 | -0.465 | -0.2918 | No |
| 62 | TPI1 |  |  | 4134 | -0.550 | -0.3513 | No |
| 63 | HDLBP |  |  | 4260 | -0.585 | -0.3691 | No |
| 64 | CITED2 |  |  | 4303 | -0.598 | -0.3707 | No |
| 65 | NFIL3 |  |  | 4309 | -0.600 | -0.3651 | No |
| 66 | HOXB9 |  |  | 4357 | -0.621 | -0.3674 | No |
| 67 | GLRX |  |  | 4374 | -0.626 | -0.3637 | No |
| 68 | SDC4 |  |  | 4412 | -0.641 | -0.3639 | No |
| 69 | ERO1A |  |  | 4434 | -0.647 | -0.3609 | No |
| 70 | TMEM45A |  |  | 4557 | -0.710 | -0.3767 | Yes |
| 71 | LDHA |  |  | 4574 | -0.722 | -0.3719 | Yes |
| 72 | ADORA2B |  |  | 4590 | -0.729 | -0.3669 | Yes |
| 73 | UGP2 |  |  | 4595 | -0.732 | -0.3597 | Yes |
| 74 | SLC2A3 |  |  | 4629 | -0.754 | -0.3579 | Yes |
| 75 | MIF |  |  | 4692 | -0.790 | -0.3613 | Yes |
| 76 | NR3C1 |  |  | 4699 | -0.796 | -0.3538 | Yes |
| 77 | TGM2 |  |  | 4837 | -0.903 | -0.3704 | Yes |
| 78 | KDELR3 |  |  | 4912 | -0.981 | -0.3740 | Yes |
| 79 | SLC2A1 |  |  | 4949 | -1.018 | -0.3698 | Yes |
| 80 | KLF6 |  |  | 4953 | -1.024 | -0.3593 | Yes |
| 81 | TPST2 |  |  | 4989 | -1.073 | -0.3544 | Yes |
| 82 | ANXA2 |  |  | 4997 | -1.087 | -0.3439 | Yes |
| 83 | PIM1 |  |  | 5002 | -1.095 | -0.3328 | Yes |
| 84 | PFKP |  |  | 5042 | -1.173 | -0.3275 | Yes |
| 85 | ACKR3 |  |  | 5047 | -1.180 | -0.3155 | Yes |
| 86 | CAVIN1 |  |  | 5050 | -1.185 | -0.3030 | Yes |
| 87 | MT2A |  |  | 5061 | -1.216 | -0.2917 | Yes |
| 88 | DUSP1 |  |  | 5090 | -1.265 | -0.2833 | Yes |
| 89 | TGFBI |  |  | 5104 | -1.305 | -0.2717 | Yes |
| 90 | P4HA2 |  |  | 5127 | -1.346 | -0.2613 | Yes |
| 91 | MT1E |  |  | 5129 | -1.359 | -0.2467 | Yes |
| 92 | MYH9 |  |  | 5132 | -1.366 | -0.2322 | Yes |
| 93 | ENO1 |  |  | 5146 | -1.410 | -0.2194 | Yes |
| 94 | LOX |  |  | 5149 | -1.418 | -0.2044 | Yes |
| 95 | BGN |  |  | 5160 | -1.486 | -0.1902 | Yes |
| 96 | SERPINE1 |  |  | 5162 | -1.501 | -0.1740 | Yes |
| 97 | S100A4 |  |  | 5189 | -1.605 | -0.1616 | Yes |
| 98 | PLAUR |  |  | 5213 | -1.721 | -0.1473 | Yes |
| 99 | TPBG |  |  | 5221 | -1.800 | -0.1291 | Yes |
| 100 | DDIT3 |  |  | 5229 | -1.853 | -0.1103 | Yes |
| 101 | F3 |  |  | 5230 | -1.856 | -0.0902 | Yes |
| 102 | EGFR |  |  | 5233 | -1.904 | -0.0698 | Yes |
| 103 | DCN |  |  | 5237 | -1.928 | -0.0495 | Yes |
| 104 | COL5A1 |  |  | 5276 | -2.605 | -0.0285 | Yes |
| 105 | CAV1 |  |  | 5281 | -2.850 | 0.0017 | Yes |
Table: GSEA details [plain text format]

  

Fig 2: HALLMARK\_HYPOXIA: Random ES distribution      
 Gene set null distribution of ES for **HALLMARK\_HYPOXIA**
